# Impact of respiratory viruses detection on outcomes in ventilated nosocomial pneumonia: an exposed/unexposed study

**DOI:** 10.1186/s13613-025-01600-6

**Published:** 2025-10-27

**Authors:** Hermann Do Rego, Julien Dessajan, Quentin Le Hingrat, Laurence Armand Lefevre, Etienne De Montmollin, Michael Thy, Stéphane Ruckly, Romain Sonneville, Lila Bouadma, Nathalie Grall, Jean-François Timsit

**Affiliations:** 1 Medical and Infectious Diseases ICU, Paris Cité University- Bichat University Hospital, Assistance Publique - Hôpitaux de Paris, Paris, France; 2 Inserm UMR 1137 - IAME Team 5 - Decision Sciences in Infectious Diseases, Control and Care INSERM/Paris Diderot, Sorbonne Paris Cité University, Paris, France; 3 Virology Department, AP-HP Nord, Bichat-Claude Bernard University Hospital, 75018 Paris, France; 4 Inserm, IAME, Université Paris Cité, 75018 Paris, France; 5 Department of Bacteriology, Paris Cité University- Bichat University Hospital, Assistance Publique - Hôpitaux de Paris, Paris, France; 6 OUTCOME REA Research Network, 93000 Drancy, France

**Keywords:** Respiratory viruses, mPCR, VAP, HAP, Coinfections, Sepsis, Pneumonia, Outcome

## Abstract

**Introduction:**

Ventilator-associated pneumonia (VAP) and ventilated hospital-acquired pneumonia (vHAP) are major causes of morbidity and mortality in intensive care unit (ICU) patients. The role of viral co-infections in these conditions is an emerging area of interest; however, their impact on clinical outcomes remains poorly understood. This study aimed to assess the effect of viral detection on mortality and other clinical outcomes in patients with bacterial vHAP/VAP.

**Materials and methods:**

We conducted a retrospective analysis of patients diagnosed with bacterial vHAP or VAP in a tertiary ICU between 2020 and 2024. All patients underwent distal respiratory sampling with quantitative culture and multiplex PCR (mPCR) testing for respiratory viruses (Biofire FilmArray Pneumonia Panel). Patients with SARS-CoV-2 infection were excluded. Those with bacterial and viral co-infections were matched 1:1 with patients having bacterial-only vHAP/VAP based on age, sex, SAPS II score, ICU admission cause, and causative bacteria. We compared clinical outcomes, including ICU mortality, 3-month mortality, ICU length of stay, and duration of mechanical ventilation between the two groups.

**Results:**

Eighty patients were included, 40 with bacterial and viral detection and 40 with bacterial-only vHAP/VAP. The median age was 63 years, and 92% of the cohort were male. Common comorbidities included diabetes (25%), heart failure (20%), chronic renal failure (20%), and chronic lung disease (32%). Nineteen percent of patients were immunocompromised. The viral pathogens identified in the co-infection group were rhinovirus/enterovirus 33% (13/40), endemic coronaviruses 30% (12/40), influenza viruses 10% (4/40), parainfluenza viruses 8% (3/10), adenovirus 8% (3/10), metapneumovirus 5% (2/40), and respiratory syncytial virus 5% (2/40). Respiratory viruses were detected in a nasopharyngeal swab in 30% (12/30). The 3-month mortality rate was 36%, ICU mortality was 32%, the median duration of mechanical ventilation was 21 days [IQR 12–31.5], and the median ICU length of stay was 24 days [IQR 13–39.5]. There were no significant differences in these outcomes between the bacterial and viral group and the bacterial-only group.

**Conclusions:**

In this cohort of patients with bacterial vHAP/VAP, the detection of respiratory viruses did not significantly impact ICU mortality, 3-month mortality, or ICU length of stay. These findings may suggest that bacterial infections are the primary determinants of clinical outcomes in vHAP/VAP.

**Supplementary Information:**

The online version contains supplementary material available at 10.1186/s13613-025-01600-6.

## Introduction

Pneumonia is the most common infection in the intensive care unit (ICU) and can present as hospital-acquired pneumonia (HAP) or ventilator-associated pneumonia (VAP). These conditions are associated with prolonged mechanical ventilation, extended ICU and hospital stays, and increased mortality [1–5].

Bacterial infections are the leading cause of nosocomial pneumonia, however, culture can be negative in some cases [6, 7]. The advent of molecular biology and multiplex polymerase chain reaction (mPCR) has led to significant advancements in the detection of respiratory viruses [8]. It is estimated that respiratory viruses, such as *Rhinovirus* or *Metapneumovirus*, are present in up to 56% of cases of community-acquired pneumonia (CAP). In the most severe cases of CAP, the presence of bacteria in association with viral infection has been shown to alter the prognosis of pneumonia, as indicated by hospital death or the need for mechanical ventilation [9, 10]. Fundamental research has shown that viral infections can facilitate and exacerbate secondary bacterial infection by altering lung epithelial integrity and the immune response [11].

In the context of HAP, several retrospective studies have reported a prevalence of respiratory viruses ranging from 5 to 34% [12–18]. However, despite these findings, the impact on patient prognosis remains inconsistent, primarily due to the lack of homogeneity across the studies. For instance, Shorr et al. included patients with non-ventilated HAP and found no significant difference in outcomes [12]. Hong et al. and Loubet et al. included patients with VAP but few patients had bacterial-viral coinfections, Loubet et al. observed a longer ICU stay in patients with bacterial-viral co-infection [13, 14]. Other studies, which included patients with both community-acquired and hospital-acquired infections, as well as those with infections involving both upper and lower respiratory tracts, reported no significant impact of viral co-infections on patient outcomes [16–18].

We therefore conducted a retrospective analysis of patients with nosocomial pneumonia requiring mechanical ventilation to determine whether the presence of respiratory viruses or absence in the lower respiratory tract was associated with worse outcomes.

## Methods

We conducted a retrospective study at the medical ICU of Bichat-Claude Bernard Hospital, a teaching tertiary referral center in Paris, France, covering the period from January 2020 to May 2024. The study included all patients aged ≥ 18 years who were admitted to the hospital for at least 48 h and diagnosed with their first episode of vHAP or VAP in the ICU. All patients were screened for Sars-Cov2 using a specific nasophayngeal PCR upon admission to the department. Patients with confirmed SARS-CoV-2 pneumonia, a history of prior nosocomial pneumonia or who objected to the use of their data for research purposes were excluded. We obtained oral consent from the participants themselves or their relatives to achieve this objective.

The trial was approved by the ethics committee of the French Intensive Care Society (CE 24–086) and the database is registered by the Commission Nationale de l’Informatique et des Libertés (CNIL, registration no 2228484v 0).

### Data collection

At ICU admission and during the ICU stay, data were collected on demographics, comorbidities, clinical examinations, laboratory and radiological findings, microbiological investigations, and therapeutic management.

**Pneumonia** was defined as:

New or worsening infiltrates on Chest X-ray or thoracic CT scan

And 

Presence of at least two of the following criteria: fever (body temperature ≥ 38.3 °C) or hypothermia (body temperature < 35 °C), dyspnea, suggestive auscultation (rales or bronchial breath sounds), purulent sputum or increased respiratory secretions, worsening gas exchange (100 mmHg decrease in PaO2/FiO2), hypotension or increasing vasopressor requirements, altered mental status, white blood cell count > 10000 cells/mm3 or < 4000 cells/mm3.

Ventilated hospital-acquired pneumonia (vHAP) was defined as an episode occurring more than 48 h after admission to a healthcare facility that requires invasive mechanical ventilation within the first 24 h after diagnosis.

Ventilator-associated pneumonia (VAP) was defined as an episode occurring 48 h after the initiation of invasive mechanical ventilation.

Clinical cure was defined as the resolution of signs and symptoms present at enrollment, such as the resolution of sepsis symptoms, improvement in oxygenation parameters indicated by a return to the inclusion PaO2/FiO2 within 50 mmHg of the initial value, and the absence of new sepsis signs. The radiographic criteria involve the resolution or lack of progression of radiological signs of pneumonia [19].

Microbiological cure was defined as the absence of the causative bacteria in respiratory samples following the completion of treatment or as a patient who, after completing therapy, remained clinically cured without relapse.

Relapse was defined as the occurrence of a new microbiologically documented pneumonia caused by the same bacteria with the same antibiotic susceptibility profile, 48 h after end of adequate antimicrobial therapy [20].

### Microbiological evaluation

vHAP or VAP was suspected based on established clinical criteria, and a distal respiratory specimen was collected, either through bronchoalveolar lavage (BAL), plugged telescoping catheter (PTC) or endotracheal aspirate (ETA). BAL, PTC and ETA were considered positive if the bacterial growth was equal or greater than 10^4^ CFU/mL (Colony-Forming Unit), 10^3^ CFU/mL or 10^5^ CFU/mL, respectively.

The Biofire FilmArray Pneumonia Panel (PN Panel; BioFire Diagnostics, LLC) was used for all specimens. This panel detects 18 bacteria (*Acinetobacter calcoaceticus-A. baumannii complex, Enterobacter cloacae complex, Escherichia coli, Haemophilus influenzae, Klebsiella aerogenes, Klebsiella oxytoca, Klebsiella pneumoniae group, Moraxella catarrhalis, Proteus spp., Pseudomonas aeruginosa, Serratia marcescens, Staphylococcus aureus, Streptococcus agalactiae, Streptococcus pneumoniae, Streptococcus pyogenes, Chlamydia pneumoniae, Legionella, pneumophila, Mycoplasma pneumoniae*), and identifies seven resistance genes (KPC, NDM, IMP, VIM, OXA-48-like, CTX-M, mecA/mecC and MREJ), and eight viruses (*Adenovirus, endemic Coronavirus, Human metapneumovirus, Human rhinovirus/enterovirus, Influenza A virus, Influenza B virus, Parainfluenza virus, Respiratory syncytial virus*) in approximately 75 min [21, 22].

In our department, all cases of suspected nosocomial pneumonia undergo this mPCR, in addition to microbiological culture.

### Definition of exposed and unexposed patients

A matched case–control study was performed. Patients presenting with a bacterial infection with evidence of a respiratory virus were considered as exposed patients (presence of a bacteria in microbiological culture or with mPCR, in conjunction with the detection of a virus with mPCR in the same respiratory sample) and compared with the unexposed patients (bacterial infections without evidence of a respiratory virus).

The exposed patients were matched with the unexposed patients according to the cause of admission (surgical vs medical), age (± 10 years), SAPSII (± 10), sex and bacteria (same genus and species, except for Enterobacterales, which were divided into 2 groups: AmpC-producing Enterobacterales vs other Enterobacterales). The matching process was exact for the 40 pairs.

### Endpoints

The primary endpoint of the study was 3-month mortality from inclusion.

The secondary endpoint included ICU mortality, hospital mortality, length of stay, duration of mechanical ventilation, clinical and microbiological cure and relapse.

### Statistical analysis

The viral-bacterial vHAP/VAP patients were compared with matched bacterial-only vHAP/VAP patients using a Wilcoxon test for paired comparisons, or a McNemar test where appropriate. All tests were two-tailed, with P < 0.05 representing statistical significance. We planned a conditional logistic regression adjusted for imbalanced parameters between exposed and unexposed patients.

Analyses were performed using R software (R Core Team (2021).

## Results

### Characteristics

**Fig. 1:**
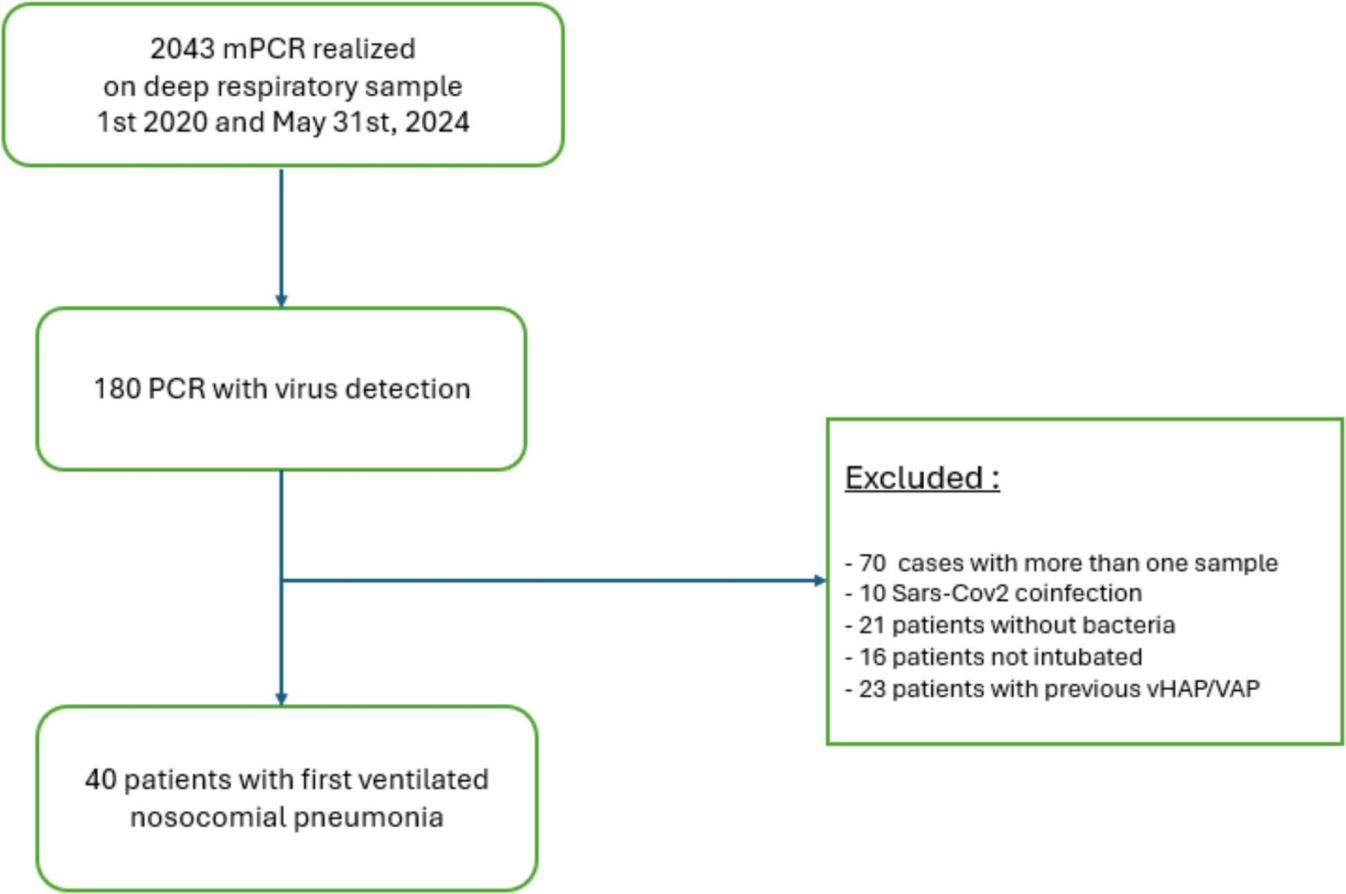
Flow chart

Between January 1st 2020 and May 31st 2024, 180 mPCR detected virus (other than Sars Cov 2) on lower respiratory tract samples. Among these, 40 patients were diagnosed with vHAP/VAP and bacterial-viral co-infection (Fig. 1), they were matched with 40 other patients on mechanical ventilation with vHAP/VAP who had an mPCR that did not detect virus.

The demographic and clinical characteristics of patients with bacterial-viral vHAP/VAP and those with bacterial-only vHAP/VAP are presented in Table 1.

**Table 1:** Clinical characteristic of 80 vHAP/VAP episodes

	Total	Viral/bacterial group (n = 40)	Bacterial group (n = 40)	*p* value
Age, years	63 [52; 70]	63 [53.5; 70.5]	63 [51; 70]	0.64
Male	74 (92%)	37 (92%)	37 (92%)	1
Height, cm	173 [169; 180]	173 [169; 179]	173 [170; 180]	0.33
Weight, kg	80.0 [69; 90]	78 [61; 87]	85 [70; 95]	**0.03**
BMI, kgs.m^−2^	25.9 [23; 30]	25.5 [21.2; 28.1]	26.1 [23.9; 31]	0.16
Diabetes	21 (26%)	10 (25%)	11 (28%)	0.78
Heart failure	18 (22%)	8 (20%)	10 (25%)	0.59
Chronic lung disease	23 (29%)	13 (32%)	10 (25%)	0.46
Chronic renal failure	16 (20%)	8 (20%)	8 (20%)	1
Stroke	11 (14%)	3 (7.5%)	8 (20%)	0.17
Liver disease	3 (3.8%)	1 (2.5%)	2 (5%)	1
Alcoholism	8 (10%)	5 (12%)	3 (7.5%)	0.71
Neoplasm	9 (11%)	6 (15%)	3 (7.5%)	0.48
Charlson score	1 [0; 2]	1 [0; 2]	1 [0; 3]	0.34
Immunocompromised	15 (19%)	9 (22%)	6 (15%)	0.42
Type of admission				0.77
Medical	65 (81%)	32 (80%)	33 (82%)	
Surgical	15 (19%)	8 (20%)	7 (18%)	
SAPS II	54.5 [44; 62.5]	52.5 [44; 64.2]	55 [43.5; 61.2]	0.91
Respiratory infection on admission	26 (33%)	12 (30%)	14 (35%)	0.8
CAP				
HAP	14	8	6	
COPD	9	3	6	
Asthma	2	1	1	
	1	0	1	
Virus detected in nasopharyngeal swab before nosocomial pneumonia	12/30 (40%)	12/19 (63%)	0/11 (0%)	** < 0.01**
*Rhinovirus/Entero*				
*Influenzae*				
*Parainfluenzae*		3		
*Coronavirus*		3		
*Adenovirus*		2		
*VRS*		1		
*Metapneumovirus*		1		
		1		
		1		
Delay between hospital admission and nasopharyngeal swab	1 [0; 3]	1 [0; 3]	1 [0; 3]	0.98
Type of nosocomial pneumonia				0.34
vHAP	26 (32%)	11 (28%)	15 (38%)	
VAP	54 (68%)	29 (72%)	25 (62%)	
Delay of nosocomial pneumoniae since onset of hospitalization	10.5 [4.8; 18]	11 [4; 17.2]	9.5 [5; 18.2]	0.8
Delay of pneumonia since intubation, days	5 [2; 11]	5 [2; 9]	6.5 [1; 12.5]	0.33
Delay between nasopharyngeal swab and pneumonia	9 [4–16]	9 [3–13]	9 [6–18]	0.66
Type of sample				0.52
BAL	42 (52%)	23 (57%)	19 (48%)	
PTC	35 (44%)	15 (48%)	20 (50%)	
ETA	3 (3.8%)	2 (5%)	1 (2.5%)	
Polymicrobial infection	24 (31%)	13 (34%)	11 (28%)	0.52
SOFA score at diagnosis	9 [6; 12]	9 [6; 11.2]	9 [6; 12]	0.51
Duration of antibacterial therapy	7 [7; 8]	7 [7; 8.5]	7 [6.5; 7]	0.09
Fever or hypothermia	35 (44%)	20 (50%)	15 (38%)	0.34
Inflammatory syndrome	53 (66%)	25 (63%)	28 (70%)	0.72
Worsening oxygenation	71 (89%)	37 (93%)	34 (85%)	0.75
New or increasing radiological infiltrates	64 (80%)	31 (78%)	33 (83%)	0.78
Tracheal secretion	60 (75%)	28 (70%)	32 (80%)	0.66
HSV reactivation in ICU	31/55 (56%)	17/30 (57%)	14/25 (56%)	0.96
CMV reactivation in ICU	16/53 (30%)	10/28 (35%)	7/25 (28%)	0.67
Associated bacteremia	8 (10%)	4 (10%)	4 (10%)	1
Associated septic shock	48 (60%)	23 (57%)	25 (62%)	0.65
Associated ARDS				
None	19 (24%)	9 (22%)	10 (25%)	
Mild	18 (22%)	11 (28%)	7 (18%)	0.34
Moderate	32 (40%)	17 (42%)	15 (38%)	
Severe	11 (14%)	3 (7.5%)	8 (20%)	
Renal replacement therapy	31 (39%)	17 (42%)	14 (35%)	0.49
Norepinephrine (> 0,5 µg/kg/min)	69 (86%)	35 (88%)	34 (85%)	0.75
Inotropes	29 (36%)	18 (45%)	11 (28%)	0.1
Extra-corporeal membrane oxygenation	25 (31%)	11 (28%)	14 (35%)	0.47

Quantitative variables are described by their median and interquartile (IQR) range. Categorical variables are presented as absolute numbers (percentages, %)

Overall, 26 patients (33%) were admitted to the ICU for respiratory infections (14 for acute community-acquired pneumonia (CAP), 9 for hospital-acquired pneumonia (HAP), 2 for severe exacerbations of chronic obstructive pulmonary disease (COPD) and 1 for asthma with bronchitis). The reason for admission to the ICU is detailed in the supplementary Table S1.

Both groups were comparable, with the exception of body weight, which was significantly higher in the bacterial-only group (85 kg vs. 78 kg, *p* = 0.03).

Pneumonia was classified as VAP in 68% and vHAP in 32% of cases. Polymicrobial infections were observed in 31% of patients. The majority of patients received a 7-day course of antibiotic therapy [7, 8]. Ten percent of pneumonias were complicated by bacteraemia. Acute respiratory distress syndrome (ARDS) according to the Berlin consensus [23] occurred in 60 (75%) patients, with mild ARDS in 22%, moderate ARDS in 40% and severe ARDS in 14%. Septic shock was present at the time of infection in 48 (60%) patients (Table 1).

mPCR was performed on nasopharyngeal swabs prior to the onset of nosocomial pneumonia in 19 cases in the bacterial/viral group yielding a positive result in 12 (63%) cases. The median delay between admission to hospital and the nasopharyngeal swab was 1 [0–3] day and the median delay between the nasopharyngeal swab and the onset of pneumonia was 9 [3–13] days. Eleven patients with only bacterial pneumonia only had a previous nasopharyngeal swab and no viruses were detected by mPCR(0/11).

Of the twelve patients in the viral/bacterial group who tested positive for a virus in their nasopharyngeal swab, all were admitted to the intensive care unit for respiratory infection. The seven other patients who tested negative for a virus were admitted for other reasons, such as cardiogenic shock or cardiac arrest.

Eleven patients in the bacterial group were given nasopharyngeal swabs for various reasons, including three cases of respiratory infection, two cases of interstitial lung disease, two cases of cardiogenic shock, two cases of cardiac surgery, one case of encephalitis, one case of polyradiculoneuritis and one case of coma.

Among the viral pathogens, rhinovirus/enterovirus was detected in 13 cases, endemic coronaviruses in 12 cases, influenza in 4 cases, parainfluenza in 3 cases, adenovirus 3 cases, metapneumovirus in 2 cases and respiratory syncytial viru4s (RSV) in 2 cases (Table 2). The incidence was highest in December, as shown in supplementary Figure S1, which presents the distribution of viral detections according to months.

**Table 2:** Microbiological documentation of 80 vHAP/VAP episodes

	Viral/bacterial group (n = 40)	Bacterial group (n = 40)	*p* value
Virus			
*Rhinovirus/enterovirus*	13	0	
Endemic coronavirus	12	0	
*Influenzae*	4	0	
*Adenovirus*	3	0	
*Parainfluenzae*	3	0	
*Metapneumovirus*	2	0	
*RSV*	2	0	
Bacteria			
*Pseudomonas aeruginosa*	9	9	
*Escherichia coli*	6	6	
Penicillinase	1	2	
AmpC	1	0	
ESBL	2	2	
*Klebsiella pneumoniae*	6	5	
ESBL	2	2	
*Staphylococcus aureus*	4	4	
MecA	1	1	
*Klebsiella aerogenes*	1	1	
*Enterobacter cloacae*	2	1	
*AmpC*	0	1	0.18^2^
*Hafnia alvei*	2	1	
*AmpC*	1	1	
*Haemophilus Influenzae*	2	2	
*Morganella morganii*	2	2	
*Proteus mirabilis*	1	1	
Penicillinase	1	0	
*Acinetobacter baumanii*	1	1	
OXA-48	0	1	
*Burkolderia spp.*	1	1	
*Stenotrophomonas maltophilia*	1	1	
*Serratia marcescens*	1	2	
*Citrobacter koseri*	0	1	
Companion bacteria^1^			
*Staphylococcus aureus*	1	1	
*Streptococcus constellatus*	1	0	
*Enterobacter cloacae*	1	1	
*Escherichia coli*	1	1	
*Enterococcus faecalis*	1	1	
*Hafnia alvei*	1	2	
*Haemophilus Influenzae*	1	0	0.6^2^
*Klebsiella oxytoca*	1	0	
*Klebsiella pneumoniae*	1	0	
*Klebsiella variicolla*	1	0	
*Neisseria meningitidis*	1	0	
*Pseudomonas aeruginosa*	0	1	
*Proteus mirabilis*	0	1	
*Streptococcus agalactiae*	0	1	
*Acinetobacter pitii*	0	1	
*Acinetobacter ursungi*	0	1	
*Citrobacter koseri*	0	1	

^1^Companion bacteria is the other bacterium documented if the culture was polymicrobial and was not taken into account during the matching process

^2^A statistical test was carried out on the following classes of bacteria: Enterobacteriaceae, AmpC-producing Enterobacteriaceae, non-fermenting Gram-negative bacilli, Staphylococcus aureus and others

Regarding the distribution of bacterial pathogens, no significant differences were observed between the study groups. The most prevalent bacterial pathogens were *Pseudomonas aeruginosa*, *Klebsiella pneumoniae*, and *Escherichia coli*. Only 3 patients (2 in case, and 1 in control group) had identification of mPCR and not with conventional culture. Detailed information is provided in Table 2.

The antibiotic therapy regimens prescribed are described in Table S2, this corresponds to documented antibiotic therapy, which is adapted to the antibiogram. The median duration of antibacterial therapy was 7 days in both groups.

### Outcomes

The analysis revealed no statistically significant differences across key outcomes. The 3-month mortality rate was 35% and 32% in control and case patients, respectively (Table 3). There was no difference in terms of length of stay or mechanical ventilation clinical and microbiological cure between bacterial/viral and bacterial only vHAP/VAP patients (Table 3).

**Table 3:** Outcomes of 80 vHAP/VAP episodes

	Viral/bacterial group (n = 40)	Bacterial group (n = 40)	*p* value
3 month-mortality	14 (35%)	15 (38%)	0.82
ICU mortality	13 (32%)	13 (32%)	0.81
Hospital mortality	13 (32%)	14 (35%)	0.64
ICU length of stay (days)	24.5 [13; 35.2]	24 [13.5; 43]	0.63
Duration of mechanical ventilation (days)	21 [12.2; 28.2]	19.5 [10; 33.5]	0.79
Duration of mechanical ventilation after infection (days)	15 [8.5; 21.5]	13 [7; 23.5]	0.91
ICU length of stay after infection (days)	16.5 [7.8; 26]	16 [9.8; 35.2]	0.32
Clinical cure	35 (88%)	36 (90%)	1
Microbiological cure	27 (68%)	23 (57%)	0.36
Relapse	9 (29%)	11 (34%)	0.65

Quantitative variables are described by their median and interquartile (IQR) range. Categorical variables are presented as absolute numbers (percentages, %)

The results were similar whether or not the virus had been recovered in a previous nasopharyngeal swab before the onset of nosocomial pneumonia (see Table S3).

## Discussion

This study aimed to assess the impact of respiratory virus detection on clinical outcomes for patients with nosocomial bacterial pneumonia requiring mechanical ventilation. Our findings might suggest that the presence of respiratory viruses does not significantly influence key clinical outcomes.

Previous studies have indicated that viral infections can facilitate bacterial co-infection by disrupting pulmonary endothelial cell function, suppressing the interferon-mediated immune response, promoting excessive inflammation, and causing dysbiosis [24–28]. These mechanisms may worsen the severity of bacterial pneumonia [29–33].

In our study, we have included patients in which a respiratory virus was detected at time of bacterial infection. However, it was not possible to distinguish whether this represented an active co-infection or a prolonged detection of viral RNA. Several studies have shown that viral shedding can last up to four weeks in some individuals [34, 35] Some studies have suggested that a low cycle threshold (CT) on quantitative PCR was indicative of active co-infection and that it correlated with higher mortality or worse outcome [36]. However, CT was not available with the technique used in this study.

In healthcare settings, respiratory viruses have the potential to spread among patients [37–41]. However, the exact mechanism by which this occurs in patients requiring mechanical ventilation remains unclear.

Unlike those for whom no virus was detected, all patients in the viral/bacterial group who had a nasopharyngeal swab and a virus detected before an episode of nosocomial pneumonia were admitted to the ICU with a respiratory infection (CAP, HAP) Patients with nosocomial bacterial pneumonia only who had a nasopharyngeal swab were admitted to the ICU for reason other than respiratory infection. These observations suggest that most patients without respiratory infection on admission potentially acquired the virus in the ICU.

Patients admitted with respiratory infections were likely to experience prolonged viral detection due to prior or superimposed bacterial nosocomial pneumonia. For patients who were not sampled prior to developing nosocomial pneumonia, the presence of the virus may indicate either nosocomial acquisition or persistence of asymptomatic viral excretion. However, without data on the presence of a virus prior to nosocomial pneumonia in all patients, or a marker of active viral infection such as cT, this remains an unprovable assumption.

Rhinovirus/enterovirus was the most commonly identified viruses in our study, consistent with the general population's viral prevalence [42–44]. These viruses are generally less pathogenic than others such as influenza, RVS or metapneumovirus. It may explain why viral detection did not affect clinical outcomes in our study. However, recent studies have shown no significant difference in hospital mortality based on the type of respiratory virus [45, 46]. Another limitation is that other respiratory viruses such as bocavirus were not tested in the mPCR we used.

Our patients are similar to a general ICU population, which included adults with relatively few comorbidities and low levels of immunosuppression (19%). Diagnosis of bacterial pneumonia was based on distal quantitative specimens and matching process adjusted for important confounders such as patients’ severity, responsible bacteria and reason for ICU stay. Pneumonia was severe in most cases, as indicated by the high incidence of septic shock and ARDS. Treatment strategies followed standard recommendations, with most patients receiving monotherapy and a median treatment duration of seven days. In addition, the risk of selection bias is limited, as all patients presenting with nosocomial pneumonia and requiring ventilation in our department undergo PCR Biofire Filmarray pneumonia testing for bacteriological documentation.

Indeed our study is a single center retrospective trial with a limited numbers of patients included. Although the matching process improves the study power, further multicenter prospective large trial is needed to provide a firm conclusion.

## Conclusion

In conclusion, our study found that the detection of respiratory viruses did not significantly impact the clinical outcomes of patients with vHAP/VAP, including mortality, ICU length of stay, or duration of mechanical ventilation.

## Supplementary Information

Additional file 1 (DOCX 28 KB)

## Data Availability

Data could be available if request to corresponding author.
