# Supplementary material for: Impact of respiratory viruses detection on outcomes in ventilated nosocomial pneumonia: an exposed/unexposed study
Source: Ann Intensive Care. 2025 Oct 27;15:172. doi: 10.1186/s13613-025-01600-6 (PMC12554854; doi:10.1186/s13613-025-01600-6)
Supplement: Supplementary file 1 — Additional file 1 (DOCX 28 KB) [file 13613_2025_1600_MOESM1_ESM.docx]

# Supplementary data

| Table S1. Reasons for ICU admission | | |
| --- | --- | --- |
|  | Viral/bacterial group (n = 40) | Bacterial group (n = 40) |
| CAP | 9 | 6 |
| Cardiogenic shock | 9 | 4 |
| Cardiac surgery | 4 | 6 |
| HAP | 2 | 5 |
| AKI | 3 | 3 |
| Cardiac arrest | 3 | 3 |
| Severe Asthma | 1 | 2 |
| AE-COPD | 2 | 1 |
| Status Epilepticus | 2 | 1 |
| Stroke | 0 | 3 |
| ILD | 0 | 3 |
| Encephalitis | 0 | 1 |
| Endocarditis | 1 | 0 |
| Colitis | 1 | 0 |
| Polyradiculoneuritis | 0 | 1 |
| Drug intoxication | 1 | 0 |
| Malaria | 0 | 1 |
| Pulmonary embolism | 0 | 1 |
| Pleural effusion | 1 | 0 |
| CAP = Community acquired pneumoniae; HAP = Hospital acquired pneumoniae ; AKI = Acute kidney injury; AE-COPD=Acute exacerbation of chronic obstructive pulmonary disease; ILD = Interstitial lung disease. | | |

Figure S1. Distribution of viral detections by month

| Table S2. Antimicrobial therapy for 80 vHAP/VAP episodes | | | |
| --- | --- | --- | --- |
|  | Viral/bacterial group (n = 40) | Bacterial group (n = 40) | *p* value |
| First antimicrobial  Amoxicillin  Cefazolin  Cefepim  Cefiderocol  Cefotaxim  Ceftarolin  Ceftazidim  Ciprofloxacin  Clindamycin  Imipenem  Levofloxacin  Linezolid  Meropenem  Oxacillin  Piperacillin  Piperacillin-tazobactam | 1  1  9  0  7  1  6  0  1  2  2  1  7  0  1  1 | 2  2  8  2  9  0  7  2  0  1  2  0  4  1  0  0 | 0.31* |
| Companion antimicrobial  0  Cefotaxim  Ciprofloxacin  Cotrimoxazole  Levofloxacin  Metronidazole  Tobramycine | 32  1  0  3  3  1  0 | 36  0  1  1  1  0  1 | 0.31 |
| * A statistical test was carried out on the following classes of antibiotics: penicillin, cephalosporins, carbapenems, quinolones, and others. | | | |

| Table S3. Outcomes according to the presence of a previous virus in a nasopharyngeal swab. | | | |
| --- | --- | --- | --- |
|  | Present (n = 12) | Absent (n = 18) | *p* value |
| Age, years | 56 [51; 64] | 67 [52; 69] | 0.3 |
| BMI, kgs.m^-2^ | 21 [19; 25] | 25 [22; 27] | 0.11 |
| Bacterial and viral detection in nosocomial pneumoniae | 12/12 | 7/18 | **<0.001** |
| SAPS II | 57.9 (17.6) | 52.7 (12.2) | 0.48 |
| 3 month-mortality | 3 (25%) | 5 (28%) | 1 |
| ICU mortality | 10 (83%) | 13 (72%) | 0.67 |
| Hospital mortality | 10 (83%) | 13 (72%) | 0.68 |
| Duration of mechanical ventilation (days) | 25 [9.8; 36] | 24 [18.8; 28] | 0.97 |
| Duration of mechanical ventilation after infection (days) | 12 [6; 28.8] | 18 [13; 24] | 0.42 |
| ICU length of stay after infection (days) | 31.5 [16; 36.5] | 28 [19.5; 36] | 0.95 |
| Clinical cure | 11 (92%) | 18 (100%) | 0.4 |
| Microbiological cure | 5 (62%) | 6 (60%) | 1 |
| Relapse | 8 (73%) | 11 (69%) | 1 |
| Quantitative variables are described by their median and interquartile (IQR) range. Categorical variables are presented as absolute numbers (percentages, %). | | | |
